# Combination of the BeWo b30 placental transport model and the embryonic stem cell test to assess the potential developmental toxicity of silver nanoparticles

**DOI:** 10.1186/s12989-020-00342-6

**Published:** 2020-03-10

**Authors:** Ashraf Abdelkhaliq, Meike van der Zande, Ruud J. B. Peters, Hans Bouwmeester

**Affiliations:** 1 Division of Toxicology, Wageningen University, P.O. box 8000, 6700 EA Wageningen, the Netherlands; 2 Wageningen Food Safety Research (WFSR), P.O. Box 230, 6700 AE Wageningen, the Netherlands; 3 Food Science and Technology Department, Faculty of Agriculture – Alexandria University, Alexandria, Egypt

**Keywords:** Silver nanoparticles, Surface chemistry, Placental transport, Embryotoxicity, Single particle-ICP-MS

## Abstract

**Background:**

Silver nanoparticles (AgNPs) are used extensively in various consumer products because of their antimicrobial potential. This requires insight in their potential hazards and risks including adverse effects during pregnancy on the developing fetus. Using a combination of the BeWo b30 placental transport model and the mouse embryonic stem cell test (EST), we investigated the capability of pristine AgNPs with different surface chemistries and aged AgNPs (silver sulfide (Ag_2_S) NPs) to cross the placental barrier and induce developmental toxicity. The uptake/association and transport of AgNPs through the BeWo b30 was characterized using ICP-MS and single particle (sp)ICP-MS at different time points. The developmental toxicity of the AgNPs was investigated by characterizing their potential to inhibit the differentiation of mouse embryonic stem cells (mESCs) into beating cardiomyocytes.

**Results:**

The AgNPs are able to cross the BeWo b30 cell layer to a level that was limited and dependent on their surface chemistry. In the EST, no in vitro developmental toxicity was observed as the effects on differentiation of the mESCs were only detected at cytotoxic concentrations. The aged AgNPs were significantly less cytotoxic, less bioavailable and did not induce developmental toxicity.

**Conclusions:**

Pristine AgNPs are capable to cross the placental barrier to an extent that is influenced by their surface chemistry and that this transport is likely low but not negligible. Next to that, the tested AgNPs have low intrinsic potencies for developmental toxicity. The combination of the BeWo b30 model with the EST is of added value in developmental toxicity screening and prioritization of AgNPs.

## Background

Engineered nanoparticles (NPs) have gained much attention in the last decades due to their unique properties compared to the corresponding bulk material, resulting in applications in a wide diversity of products [1–3]. Silver nanoparticles (AgNPs) are used in very diverse consumer related products such as textile, toys, and food containers, where they are generally applied because of their antimicrobial properties [4, 5]. This widespread use has increased the potential for human exposure to AgNPs [6].

Humans can be exposed to various forms and types of AgNPs resulting from their release during a product’s life cycle [7]. AgNPs with different chemical surface modifications are currently being explored to find the optimum between maximal functionality for the application balanced against minimal toxicological hazards, which is known as the Safe-by-Design approach [8, 9]. Upon release of AgNPs into the environment, they are susceptible to sulfidation processes resulting in silver sulfide nanoparticles (Ag_2_S NPs), which are considered the main form of particulate silver in the environment [10]. In addition, in vivo systemically available AgNPs or ionic silver have also been shown to transform into Ag_2_S NPs [11]. Direct use of Ag_2_S NPs has also been described in engineering and biomedical applications [10, 12]. Ag_2_S NPs have been shown to be stable in soils resulting in a potential uptake and accumulation of Ag_2_S NPs in plants and the human food chain [13]. In our studies, we therefore not only included AgNPs with different chemical surface modifications, but also Ag_2_S NPs.

Previously, we studied the potential transport of AgNPs with different surface modifications across monolayers of intestinal epithelial cells in vitro [14]. We showed that in vitro simulated human digestion had a drastic effect on the dissolution of AgNPs, an effect that was surface coating dependent. A concentration dependent cellular uptake and/or association, albeit low, was observed. Also available sub-chronic data from rodent studies indicate systemic uptake and retention of Ag in tissues [11, 15]. Therefore, in the current work we assessed whether AgNPs would also be able to pass the placental barrier and if so, to what extent. In addition, to obtain further insight in possible hazards for the developing fetus, the in vitro developmental toxicity of the AgNPs was investigated using the mouse embryonic stem cell test (EST).

The placental barrier has received special consideration in the field of toxicology as fetal exposure to NPs might be associated with reduced fetal growth and embryotoxicity [16–19]. NPs uptake and transport across the placental barrier has been reported to be dependent on the surface chemistry, size, and the chemical composition of the NPs [20, 21]. To date, several alternative in vitro and ex vivo models have been developed and used to study the transport of NPs across the human placental barrier, i.e. ex vivo placental perfusion and in vitro models using primary cells, and cell lines [18, 22, 23]. BeWo b30 placental cell layer has been used as a model to study the barrier for maternal-fetal exchange [24, 25]. This model is considered an easy and robust screening method to predict the placental transfer of xenobiotics, nutrients, compounds, and NPs [26–28]. The BeWo b30 cells form a polarized cell layer and express placental differentiation markers [27, 29–31]. Additionally, the BeWo b30 cell layer model has been optimized for nanoparticle transport studies using fluorescent polystyrene NPs (PSNPs) [16]. The human silver concentration in blood is usually below 1 μg/L, but can reach levels of up to 194 μg/L in individuals with a compromised skin treated with silver containing creams [32–34]. In our in vitro study we exposed layers of BeWo b30 cells to a AgNP concentration of 1 mg/L.

Combining the BeWo cell layer model with the EST has proven to be a way to predict relative in vivo developmental toxicity [35–37]. The EST has been validated by the European Centre for the Validation of Alternative Methods (ECVAM) [38, 39], where the differentiation of the mouse embryonic stem cells (mESCs) into beating cardiomyocytes is representing the early stages of embryonic development [40, 41]. Only few studies used the EST to examine the developmental toxicity of AgNPs [41].

This study aims to determine the potential prenatal developmental toxicity of pristine AgNPs with different surface chemistries compared to the ‘aged’ AgNPs and AgNO_3_. For this, a combination of the BeWo b30 placental transfer model and the EST was used. Uptake/association and transport of ionic silver and AgNPs across the placental cell layer were determined using inductively coupled plasma mass spectrometry (ICP-MS) and single particle (sp) ICP-MS, respectively. Confocal imaging was used to assess cellular penetration of AgNPs into the differentiated cardiomyocytes.

## Results

### Physicochemical characterization of the AgNPs suspensions and AgNO_3_ solution

Four AgNPs were used in this study: lipoic acid-coated (LA), citrate-coated (Cit), branched polyethylenimine-coated (BPEI) AgNPs, and silver sulfide nanoparticles (Ag_2_S) NPs that are regarded as aged AgNPs [10]. The hydrodynamic sizes of all the AgNPs were measured in nano-pure water at room temperature using DLS (Table 1). Only the (BPEI) AgNPs were positively charged, while the other AgNPs were negatively charged. All the AgNPs suspensions and the AgNO_3_ solution, prepared in DMEM^+^, were analyzed immediately after preparation to quantify the total silver content and the fraction of silver in particulate form using ICP-MS and spICP-MS, respectively (Figure S1). In addition, sp-ICP-MS was used to characterize the suspensions of AgNP, the results are presented in Fig. 4 and explained along with the results of the transport study across the layer of BeWo cells. The sp-ICP-MS characterization in the cell culture medium used for the EST can be found in the supplementary information (Figure S2).

**Table 1:** Physicochemical characteristics of AgNPs

	Size (TEM) nm ± SD^a,c^	Hydrodynamic size (DLS) nm ± SD^a^	ζ- potential (mV) ± SD^a,c^	Dissolution (%) ± SD^b^	
				0 h	120 h
*(LA) AgNPs*	51 ± 5	70 ± 8	−54 ± 3	15 ± 4	32 ± 2^#^
*(Cit) AgNPs*	48 ± 5	61 ± 5	− 46 ± 1	25 ± 3	31 ± 1
*(BPEI) AgNPs*	47 ± 5	62 ± 1	+ 73 ± 1	17 ± 1	29 ± 2^#^
*Ag*_*2*_*S NPs*	28 ± 20	201 ± 28	−22 ± 1	31 ± 1	25 ± 6
*AgNO*_*3*_	–	–	–	–	–

^a^Measured in nano-pure water

^b^Measured in complete cell culture medium (DMEM^+^)

^c^ Provided by Nanocomposix Inc.

^#^ Significant difference between 0 and 120 h incubation within the same AgNPs (*p*-value < 0.05)

Upon preparing the (1 mg/L) AgNPs suspensions and AgNO_3_ solution (t = 0), the percentages of dissolution of (LA), (Cit), and (BPEI) AgNPs in the DMEM^+^ were between 15 and 25%, while in the Ag_2_S NPs suspension it was 31%. The AgNO_3_ solution did not contain detectable levels of AgNPs. The dissolution of the AgNPs in DMEM^+^ was measured following a maximal incubation of 120 h which represents the total exposure time in the mESCs differentiation assay (Table 1). For the (LA) and (BPEI) AgNPs suspensions, the percentage of dissolution of AgNPs increased over these 120 h by ~ 2 fold, while for (Cit) AgNPs and Ag_2_S NPs no significant difference in the percentage of dissolved AgNPs between the two time points was detected (Table 1).

### Cytotoxicity assessment of AgNPs and AgNO_3_ in BeWo b30 cells

**Fig. 1:**
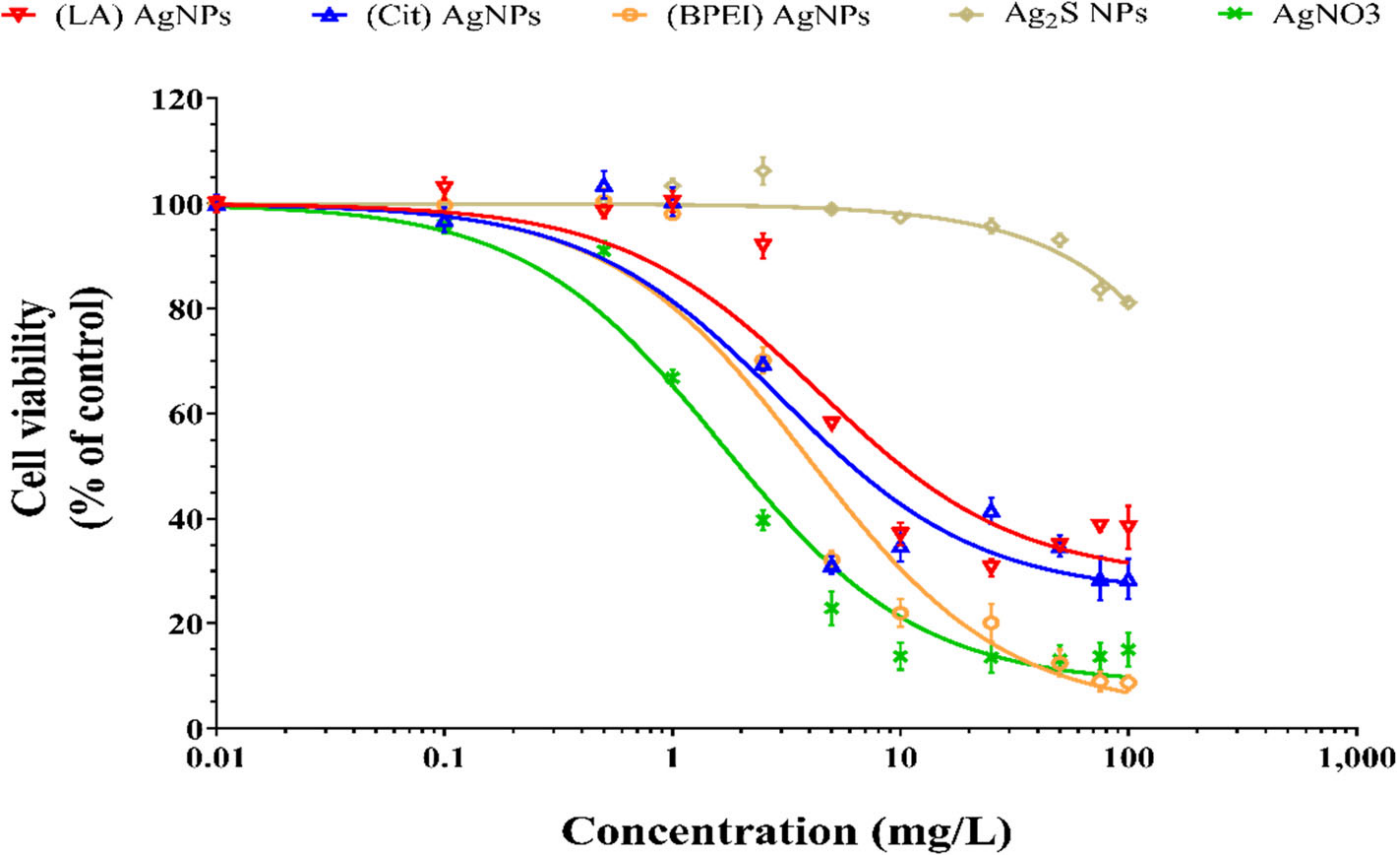
Concentration dependent effect of (LA), (Cit), (BPEI) AgNPs, Ag_2_S NPs, and AgNO_3_ on the viability of BeWo b30 cells after 24 h exposure quantified using the ATPlite viability assay. Viability is given as a percentage of the control (mean ± SD; *n* = 3)

The viability of BeWo b30 placental cells was assessed upon 24 h exposure to a concentration series of AgNPs suspensions or AgNO_3_ solutions (Fig. 1). Exposure to AgNO_3_ showed the highest cytotoxicity with an IC_50_ of 2 mg/L, while exposure to Ag_2_S NPs resulted in substantially less cytotoxicity with an IC_50_ > 100 mg/L (Table 2). For the subsequent BeWo b30 cell layer exposure studies, a non-toxic concentration of the AgNPs was used (1 mg/L). Following the 24 h exposure to 1 mg/L for AgNO_3_, the cell viability was decreased to 67%.

**Table 2:** The IC_50_ values of AgNPs and AgNO_3_ in BeWo b30 placental cells after 24 h exposure

Treatment	IC_50_ (mg/L)
(LA) AgNPs	11
(Cit) AgNPs	6.2
(BPEI) AgNPs	4.3
Ag_2_S NPs	> 100
AgNO_3_	2

*IC*_*50*_ the concentration where 50% of BeWo b30 cells are viable

### Cellular uptake/association and transport of AgNPs and AgNO_3_ through the BeWo b30 placental barrier

For the uptake/association and transport studies cell layer of BeWo b30 placental cells were used after 6 days of differentiation. Good integrity was confirmed by TEER values ranging between 80 and 100 Ω.cm^2^ (as defined by others [31]) and low transport values of three paracellular transport marker compounds (i.e. 8, 6, 7% for LY, and 4 kDa and 10 kDa FITC-dextrans, respectively).

**Fig. 2:**
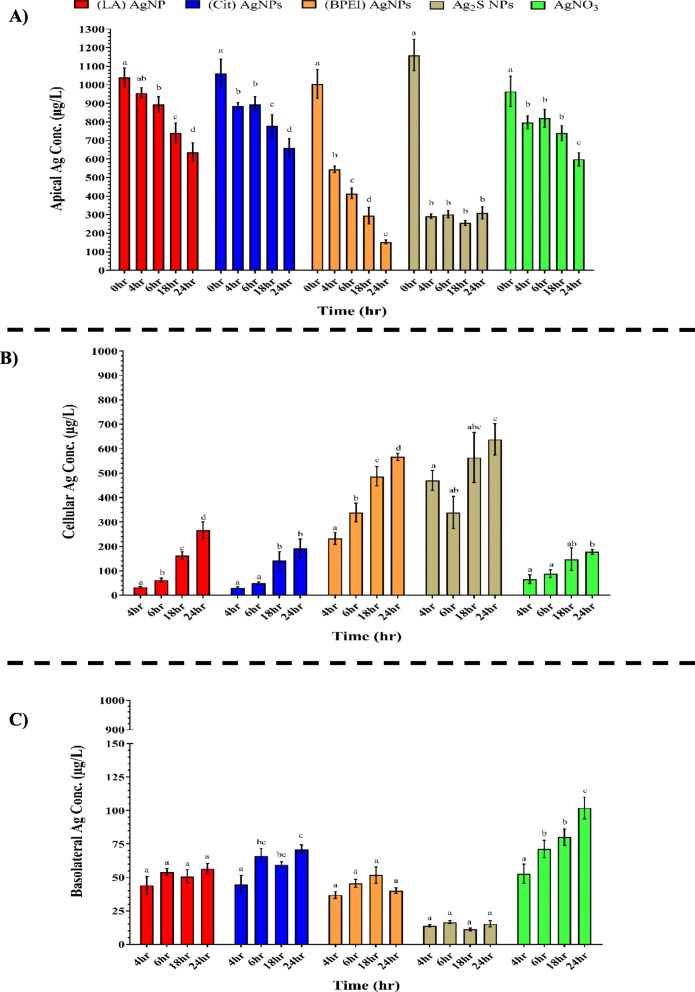
Total silver content in the **a** apical, **b** cell lysate, and **c** basolateral compartments of the BeWo b30 placental transfer model after 4, 6, 18 and 24 h exposure to 1 mg/L of (LA), (Cit), (BPEI) AgNPs, Ag_2_S NPs, or AgNO_3,_ measured using ICPMS. Concentrations are given as the mean ± SD (n = 3). Values with different letters are significantly different within the same treatment (*p* ≤ 0.05)

**Fig. 3:**
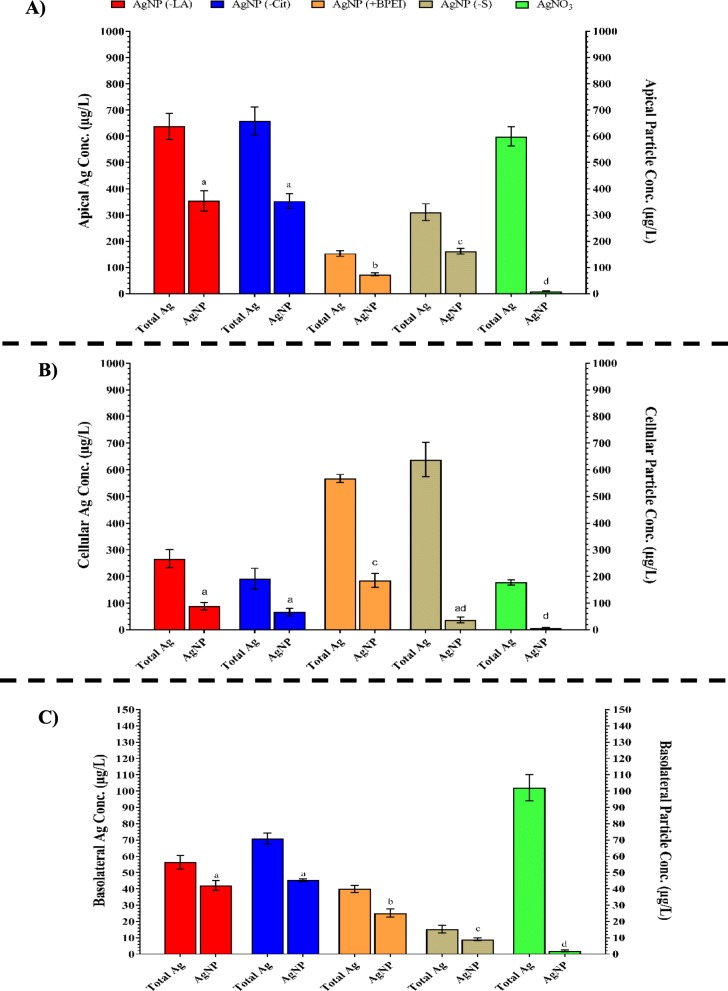
Total silver content (on the left axis) versus the AgNPs content (mass-based on the right axis) in the **a** apical, **b** cell lysate, and **c** basolateral compartments of the BeWo b30 placental transfer model after 24 h exposure to 1 mg/L of (lA), (Cit), (BPEI) AgNPs, Ag_2_S NPs, or AgNO_3_, measured using spICP-MS. Concentrations are given as the mean ± SD (n = 3). Values with different letters are significantly different within the same treatment (*p* ≤ 0.05)

The BeWo b30 cell layer were exposed for 4, 6, 18, and 24 h to 1 mg/L of the different AgNPs suspensions, or the AgNO_3_ solution. The silver content in the media from the apical, cellular and basolateral compartments was quantified using ICP-MS measurements at all exposure times and expressed as total Ag (i.e. ionic silver and particulate silver; Fig. 2). In addition, the silver fraction as AgNPs was quantified using sp-ICP-MS measurements, but only after 24 h exposure (Fig. 3). The TEER measurements performed after the exposure to the AgNPs and AgNO_3_ indicated that the BeWo b30 barrier integrity was not affected.

The total Ag content in the apical compartment declined significantly in a time-dependent manner in the (LA), (Cit), (BPEI) AgNPs and AgNO_3_ exposure groups (Fig. 2a). In the Ag_2_S NPs exposure group, the total Ag content of the apical compartment declined rapidly at early time points but remained stable from 4 h exposure onwards. This early decline was significantly larger compared to the decline observed for the other AgNPs. The fractions of total Ag present as AgNPs in the apical compartments upon 24 h exposure to (LA), (Cit), (BPEI) AgNPs, and Ag_2_S NPs were comparable (Fig. 3a; Table 3). The fraction of AgNPs was lower for the AgNO_3_ group.

**Table 3:** The fraction of AgNPs as % of total Ag content in the apical, cellular and basolateral compartments of the BeWo b30 placental cell model after 24 h exposure as calculated from the data presented in Fig. 3

Treatment	Compartment		
	Apical	Cellular	Basolateral
(LA) AgNPs	55%	33% ^*^	77% ^* #^
(Cit) AgNPs	54%	35% ^*^	64% ^#^
(BPEI) AgNPs	52%	32% ^*^	63% ^#^
Ag_2_S NPs	48%	6% ^*^	59% ^#^
AgNO_3_	2%	4% ^*^	2%

* Significant difference compared to the apical compartment of the same exposure group (*p*-value < 0.05)

^#^ Significant difference between cell and basolateral compartments of the same exposure group (*p*-value < 0.05)

For the cellular compartment of the BeWo b30 cell model, the total Ag content increased significantly in a time-dependent manner for the (LA), (Cit) and (BPEI) AgNPs, except for the 6 h time point where the increase versus the 4 h time point was not statistically significant (Fig. 2b). While the time-dependent increase in the total Ag content was less pronounced upon exposure to Ag_2_S NPs or AgNO_3_, the total Ag concentration in the cellular compartment increased significantly after 24 h exposure compared to that observed after 4 and 6 h exposure. In the cellular compartment, the concentration of AgNPs upon 24 h exposure to (BPEI) AgNPs was higher compared to what was observed following exposure to the other AgNPs and AgNO_3_ (Fig. 3b). However, upon expressing the AgNPs concentrations in the cellular compartment after 24 h exposure as percentages of the total Ag present in the cellular compartments, these percentages between (LA), (Cit), and (BPEI) AgNPs were comparable. Upon exposure to Ag_2_S NPs, the cellular compartment contained a lower percentage of AgNPs (Fig. 3b, Table 3). The cellular compartment exposed to AgNO_3_ contained only 4% of AgNPs of the total Ag content in this compartment (Fig. 3b, Table 3).

In spite of the time-dependent decrease in the amount of total Ag in the apical compartment and an increase in the amount of total Ag in the cellular compartment (Fig. 2a and b), no time-dependent transport of total Ag across the cell layer to the basolateral compartment was observed upon exposure to all the AgNPs, apart from incidental significant differences for the (Cit) AgNPs (Fig. 2c). Upon AgNO_3_ exposure, the total Ag content in the basolateral compartment showed a time-dependent increase. Additionally, AgNPs were detected in the basolateral compartment. The highest percentage of AgNPs from the total Ag content was detected in the basolateral compartments exposed to the (LA) AgNPs followed by (Cit) and (BPEI) AgNPs and Ag_2_S NPs (Fig. 3c and Table 3). Following exposure to AgNO_3_ only 2% of the compartment’s total Ag content was present as AgNPs.

### Size distribution of AgNPs before and after BeWo b30 exposure

**Fig. 4:**
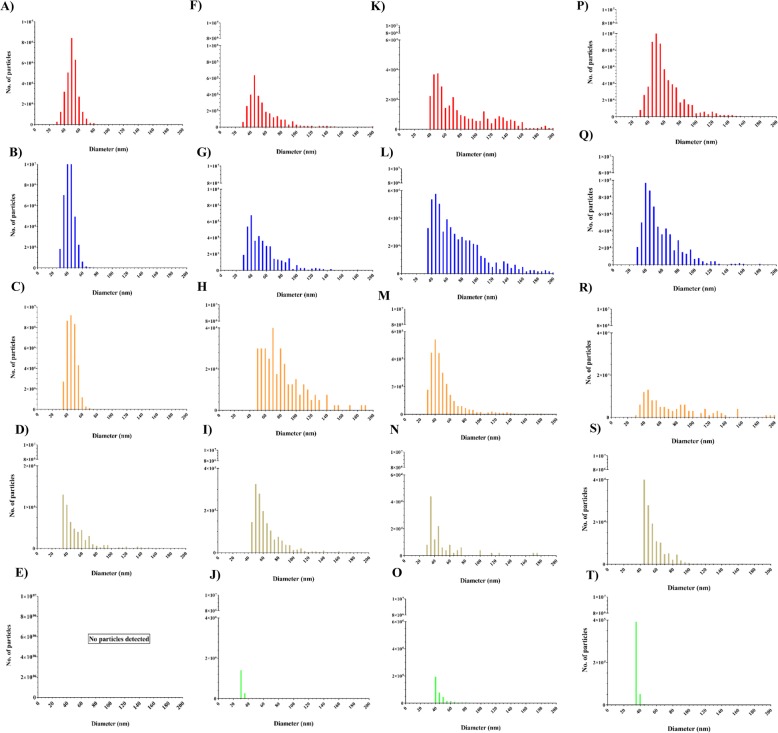
Number-weighted size distributions of AgNPs generated by spICP-MS measurements of the suspensions (1 mg/L) of: **a** (LA) AgNPs, **b** (Cit) AgNPs, **c** (BPEI) AgNPs, **d** Ag_2_S NPs, and **e** AgNO_3_. Number-weighted size distributions of AgNPs in the apical compartments of the BeWo b30 monolayer model upon 24 h exposure to 1 mg/L of: **f** (LA) AgNPs, **g** (Cit) AgNPs, **h** (BPEI) AgNPs, **i** Ag_2_S NPs, and **j** AgNO_3_. Number-weighted size distributions of AgNPs in the cellular compartments of the BeWo b30 monolayer model upon 24 h exposure to 1 mg/L of: **k** (LA) AgNPs, **l** (Cit) AgNPs, **m** (BPEI) AgNPs, **n** Ag_2_S NPs, and **o** AgNO_3_. Number-weighted size distributions of AgNPs in the basolateral compartments of the BeWo b30 monolayer model upon 24 h to 1 mg/L of: **p** (LA) AgNPs, **q** (Cit) AgNPs, **r** (BPEI) AgNPs, **s** Ag_2_S NPs, and **t** AgNO_3_

The size distributions of the AgNPs were assessed in the different compartments (apical, cellular, and basolateral) of the BeWo b30 model. The sp-ICP-MS data were transformed into size distribution plots depicting the number of particles corresponding to the size, clustered in 5 nm diameter clusters (Fig. 4). The size distributions of the (LA), (Cit), and (BPEI) AgNPs exposure suspensions were comparable, with median particle sizes of 50, 45, and 45 nm, respectively (Fig. 4a, b and c). The size distribution of Ag_2_S NPs suspension demonstrated a right-skewed size distribution with a median particle size of 35 nm (Fig. 4d). No AgNPs were detected in the AgNO_3_ solution (Fig. 4e).

After 24 h exposure, the total number of AgNPs that was detected in the apical compartments decreased for all four AgNPs (Fig. 4f-i) where the (BPEI) AgNPs (Fig. 4h) showed the largest decrease. The size distributions of all the AgNPs in the apical compartment were right skewed. The size distributions of (LA) and (Cit) AgNPs were the least affected with a median size of 45 and 40 nm, respectively (Fig. 4f and g). The size distribution of both the (BPEI) AgNPs and Ag_2_S NPs indicated an increased median size of 70 nm and 50 nm, respectively, indicating agglomeration of the particles. (Fig. 4h and i). Some AgNPs were detected in the AgNO_3_ sample with a median size of 30 nm (Fig. 4j).

The size distributions of all AgNPs in the cellular compartments were also right skewed. This was more pronounced than in the apical compartments for the (LA) and (Cit) AgNPs (Fig. 4k and l), with median sizes of 50 and 45 nm, respectively. It was less pronounced for the (BPEI) AgNPs and Ag_2_S NPs (Fig. 4m and n), with median sizes of 40 nm and 35 nm, respectively. The AgNPs that were detected in the cellular compartment upon exposure to AgNO_3_ had a median size of 40 nm (Fig. 4o).

The size distributions of all AgNPs in the basolateral compartments were right skewed and were very similar in shape but different in number of particles. The (LA) and (Cit) AgNPs were less right skewed than in the cellular compartments and had median sizes of 50 and 40 nm, respectively (Fig. 4p and q). The size distribution curve of (BPEI) AgNPs was very wide, with a median size of 45 nm (Fig. 4r). The Ag_2_S NPs had a median size of 45 nm and featured a size distribution pattern very similar to that in the starting suspension (Fig. 4s and d). For AgNO_3,_ AgNPs with median size of 35 nm were detected (Fig. 4t).

### In vitro developmental toxicity assessment of AgNPs and AgNO_3_

**Fig. 5:**
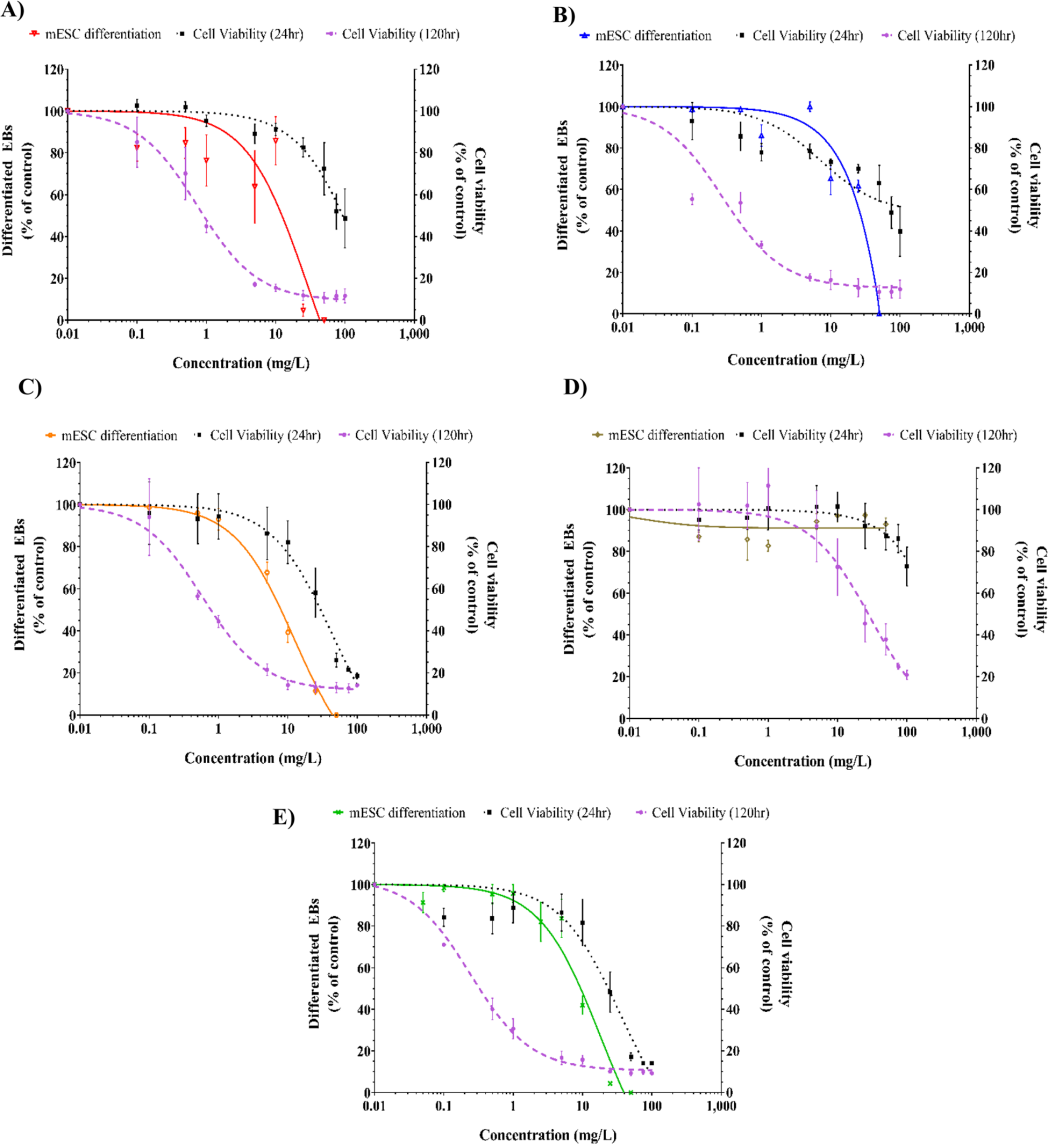
Concentration–response curves for cytotoxicity towards mESCs and for the effect on differentiation into contracting cardiomyocytes of: **a** (LA) AgNPs, **b** (Cit) AgNPs, **c** (BEPI) AgNPs, **d** (Ag_2_S) NPs, and **e** AgNO_3._ The viability of mESCs (right y-axis) was assessed using the ATPlite assay after 24 h and 120 h of exposure. The differentiation of mESCs into contracting cardiomyocytes (left y-axis) was scored after microscopical evaluation. Values are given as a percentage of the control (mean ± SD; n = 3)

To assess the potential in vitro developmental toxicity of AgNPs and AgNO_3_, the EST was employed. First, the viability of the mESCs was assessed upon 24 and 120 h exposure to a concentration series of AgNPs and AgNO_3_ using the ATPlite assay (Fig. 5). The Ag_2_S NPs showed lowest toxicity after 24 and 120 h exposure with an IC_50_ of > 100 and 29 mg/L, respectively, while AgNO_3_ showed the highest toxicity after 24 and 120 h exposure with an IC_50_ of 25 and 0.33 mg/L, respectively (Table 4). All tested AgNPs and AgNO_3_ induced a concentration-dependent reduction in viability of the mESCs with higher cytotoxicity after 120 h compared with 24 h exposure.

**Table 4:** The IC_50_ and ID_50_ of AgNPs and AgNO_3_ after 24 and 120 h exposure in mESCs

	IC_50_ (mg/L) (24 h)	IC_50_ (mg/L) (120 h)	ID_50_ (mg/L)	Calculated concentration of dissolved Ag^+^ (mg/L) in ID_50_ (120 h)	Calculated % of inhibition of differentiation induced by dissolved Ag^+^
(LA) AgNPs	100	0.9	13	4.16	22.5%
(Cit) AgNPs	100	0.38	16	4.96	31%
(BPEI) AgNPs	30	0.8	8	2.24	16%
Ag_2_S NPs	> 100	29	> 100	25	N.A.
AgNO_3_	25	0.33	9.5	–	–

*IC*_*50*_ the concentration where 50% of mESCs are viable

*ID*_*50*_ the concentration of 50% inhibition of differentiation of EBs into beating cardiomyocytes

*N.A.* Not applicable

**Fig. 6:**
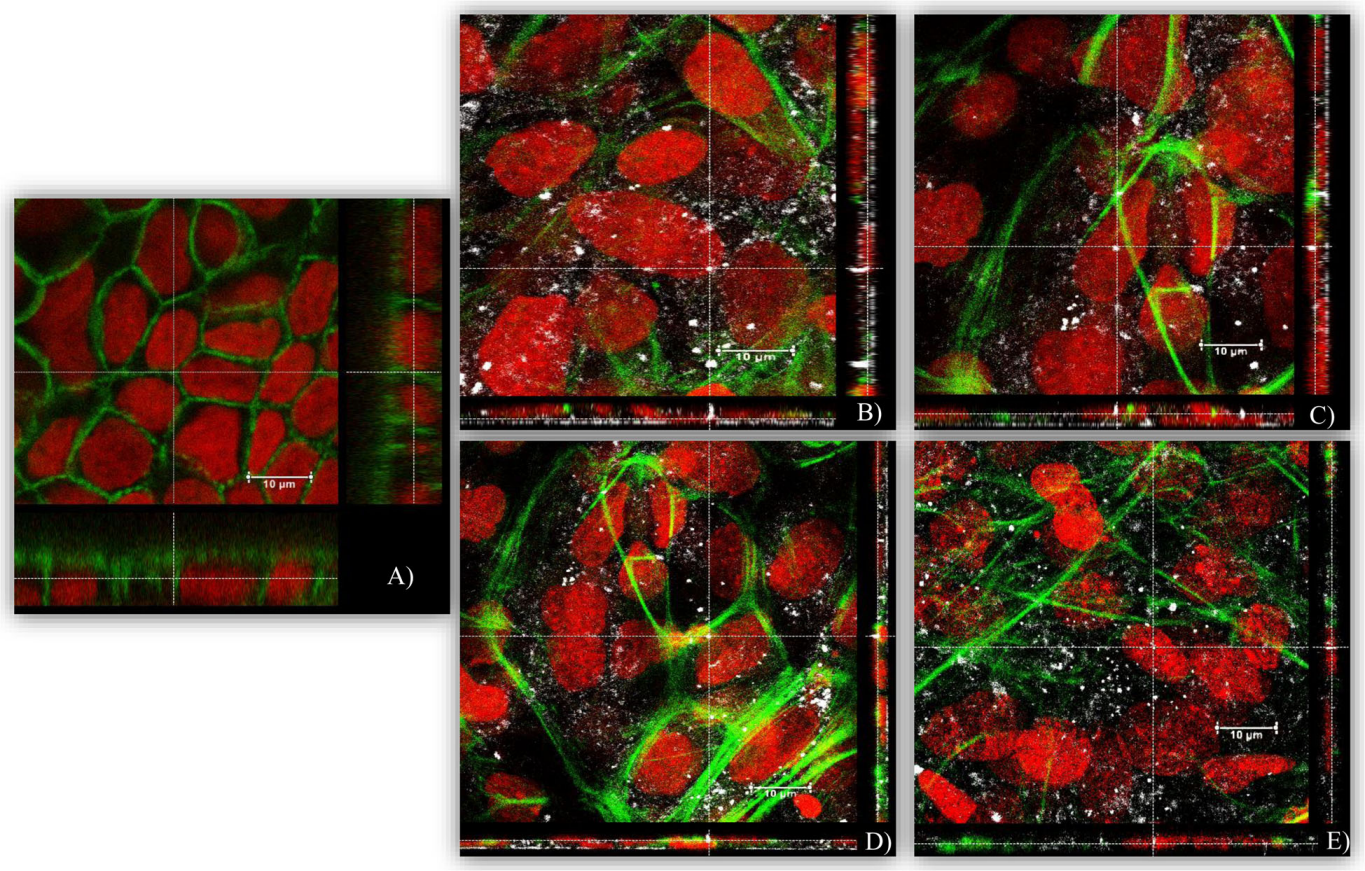
Confocal microscopic images of differentiated cardiomyocytes after 10 days of differentiation. **a** Negative control, **b** cells exposed to 1 mg/L (LA) AgNPs, **c** (Cit) AgNPs, **d** (BEPI) AgNPs and **e** Ag_2_S NPs. Nuclei were stained in red (RedDot-2), actin was stained in green (Alexa − 488 Phalloidin), and AgNPs are shown in white (back scatter)

Subsequently, the potential inhibitory effects of AgNPs and AgNO_3_ on the differentiation of mESCs into contracting cardiomyocytes was studied. Except for the Ag_2_S NPs, all tested AgNPs and AgNO_3_ induced a concentration-dependent inhibition of the differentiation of the mESCs into contracting cardiomyocytes (Fig. 5). The (BPEI) AgNPs were the most potent to inhibit the differentiation of mESCs with an ID_50_ of 8 mg/L The AgNO_3_, (LA) and (Cit) AgNPs had ID_50_ of 9.5, 13 and 16 mg/L, respectively, while the ID_50_ of Ag_2_S NPs was > 100 mg/L (Table 3). For all AgNPs and AgNO_3_, the inhibitory effects on the mESCs differentiation were observed at higher concentrations than those associated with the decrease in mESCs viability after 120 h exposure, as indicated by a higher ID_50_ than IC_50_. This indicates that effects on mESCs differentiation are likely due to cytotoxicity. In order to estimate the influence of Ag ions, released from dissolving AgNPs, on the mESCs differentiation we interpolated the concentration of Ag ions present at the ID_50_ for each AgNPs (Table 3). The concentration of Ag ions present at the ID_50_ was estimated to account for 16, 23, and 31% of the observed inhibition of the mESCs differentiation upon exposure to (BPEI), (LA), and (Cit) AgNPs at their ID_50_, respectively. In case of Ag_2_S NPs, no inhibition of the mESCs was found up to the highest concentration tested of the Ag_2_S NPs. Confocal imaging demonstrated that AgNPs were internalized into the differentiated cardiomyocytes. AgNPs were mainly localized in the cytoplasm and to some extent in the nucleus (Fig. 6).

## Discussion

This study aimed to investigate the potential prenatal developmental toxicity of pristine AgNPs with different surface chemistries and of aged Ag_2_S NPs. For this, we combined the BeWo b30 placental transport model with the EST. This enabled us to evaluate the potential fetal exposure to AgNPs and to study the likelihood of AgNPs to induce in vitro developmental toxicity.

### The interaction of ag and AgNPs with BeWo b30 placental cells

Exposure to the (LA), (Cit), and (BPEI) AgNPs and AgNO_3_ showed a concentration-dependent decrease in BeWo b30 cell viability. The positively charged (BPEI) coated AgNPs were more toxic, especially at concentrations higher than 1 mg/L, compared to the negatively charged (LA) and (Cit) coated AgNPs. In line with this observation, higher cytotoxicity for positively charged versus negatively charged NPs in BeWo b30 cells has been reported previously for polystyrene NPs [42]. Additionally, it has been reported that oleate coated ferric oxide NPs were more toxic to BeWo b30 cells than uncoated ones [26], indicating that both the surface charge and surface chemistry can cause differences in toxicity.

Exposure of the placental BeWo cell layer to 1 mg/L of (LA), (Cit), (BPEI) AgNPs, and AgNO_3_ for different durations resulted in a time-dependent increase of total Ag in the cellular compartments. The transport of silver across the placental cell layer was favorable as ionic silver rather than particulate, which was similarly reported using the ex vivo human placental perfusion model where the transport of ionic silver was ~ 10-fold higher than that of particulates [43].

The spICP-MS measurements after 24 h exposure to the different AgNPs showed the presence of AgNPs in the cellular and basolateral compartments. The surface chemistry of the AgNPs significantly affected the detected concentration of AgNPs in the cellular compartment where the highest concentrations of AgNPs were observed following exposure to (BPEI) AgNPs followed by (LA) AgNPs, (Cit) AgNPs and Ag_2_S NPs. A comparable effect of the surface chemistry of NPs on the cellular uptake/association was also reported for ferric oxide NPs where oleate coated NPs were significantly lower associated with the BeWo cell layer than uncoated NPs [26]. Besides, the cellular compartment contained a lower AgNPs percentage (of the total Ag content) compared to the AgNPs percentage in the exposure concentration in the apical compartment. This suggests a preference towards cellular uptake/association of ionic silver over AgNPs or possible dissolution of the AgNPs cellularly following their uptake [44–46].

In the basolateral compartments of all the exposure groups, AgNPs were detected. In general, the AgNPs percentage (of the total Ag content) in the basolateral compartment was higher than the AgNPs percentage (of the total Ag content) in the cellular compartment. This could suggest a preference towards cellular excretion of AgNPs over ionic silver. Also, the AgNPs measured could result from de novo formation of AgNPs from ionic silver that might reflect a tendency of the ionic silver to form these new particles in the cell culture medium more than the cellular environment. This later scenario is likely to happen taking into consideration the presence of AgNPs in all compartments upon exposure to AgNO_3_.

### Contribution of surface chemistry of AgNPs on transport across a layer of BeWo cells

The surface chemistry of the AgNPs significantly affected the detected concentration of AgNPs also in the basolateral compartment where the highest concentrations of AgNPs were observed following exposure to (LA) AgNPs and (Cit) AgNPs followed by (BPEI) AgNPs and Ag_2_S NPs. The surface chemistry of NPs was reported to play a role in the transport of ferric oxide NPs where the placental transport was significantly higher for oleate coated NPs than uncoated NPs [26]. Also, the placental transport of pegylated AuNPs was reported to be higher than that of sodium carboxylated AuNPs of the same size (Aengenheister et al. 2018a).

Next to the surface chemistry of NPs, their size [20, 23] and composition [24] are also suggested to influence their transport through the placental barrier model. The transport of PSNPs was described to be size dependent, where transport of 50 nm PSNPs was six times higher than that of 100 nm PSNPs [16]. The placental transport of AuNPs also showed a size-dependency, where 10 to 30 nm AuNPs did not cross the placental barrier [47] and the smaller 3 and 4 nm AuNP crossed the placental barrier [21]. Upon the exposure of either the BeWo b30 (up to 24 h) or the perfused human placenta (up to 6 h) to 25 and 50 nm silica NPs, no placental transport could be detected [24]. Following exposure of perfused human placenta to AgNPs with different surface chemistries (polyethylene glycol and sodium carboxylate, and a primary size of 7–15 nm) very low translocation has been observed. In the fetal circulation 0.02 to 0.2% of the administered dose was detected as total silver in the fetal circulation. Only a small mass fraction of this silver was detected in particulate form (> 25 nm). Interestingly, the authors point out that AgNPs in the fetal circulation could originate from de novo formation following ionic Ag translocation [43]. From the limited number of in vivo studies of placental transport of NPs where pregnant animals were used, AgNPs were found to be able to cross the placental barrier of rats and reach the fetus [48–52]. The study of Fennell et al. reported that 24 h after intravenous administration to pregnant rats with AgNPs, about 3–4%, measure as total Ag, of the administered dose was found in the fetus [48]. The transported levels that we observed ranged between 1 and 8% as total Ag and between 1 and 5% as AgNPs and are thus in between the observations in rodents and the human placenta perfusion studies.

### Potential fetal toxicity following exposure to AgNPs and AgNO_3_

Following the capability of the AgNPs and AgNO_3_ to cross the BeWo b30 cell layer in vitro either as ionic or as particulate silver, induction of adverse outcomes upon reaching the embryo cannot be excluded. We therefore assessed the potential developmental toxicity of the AgNPs using the EST. The EST represents a good tool to measure the developmental toxicity in vitro as it has been validated by ECVAM and it showed a good level of concordance in the in vitro to in vivo comparisons [53, 54]. Combining the EST with the BeWo placental transport model even resulted in an increased predictability of in vivo developmental toxicity of chemicals [37].

Our data from the EST did not point towards potential developmental toxicity because the effects of the AgNPs and AgNO_3_ on differentiation of mESCs into contracting cardiomyocytes were only observed at cytotoxic concentrations [39]. These result are in line with the observations of Park et al. and Corradi et al. where 7, 20, 80, and 113 nm AgNPs induced dose-dependent cytotoxicity, and inhibition of mESCs differentiation only at concentrations higher than the ones associated with cytotoxicity [53, 55]. Besides, the IC_50_ values for cytotoxicity of the AgNPs in the mESCs were higher than what would be considered realistic in vivo concentrations as our results from the BeWo b30 transport experiments indicated low transport of AgNPs into the fetal compartment. Additionally, considering the oral route as one of the main exposure routes of the AgNPs, it is important to take into account also their very low transport across the intestinal barrier in vitro [14, 56] and in vivo [15] which will consequently lower the concentrations of the AgNPs that might reach the fetus.

### Effects following exposure to aged AgNPs

It is of interest to note that the aged silver sulfide nanoparticles showed a different behavior compared to the pristine AgNPs. Although the dissolution and charge of the Ag_2_S NPs were comparable to those of the (LA) and (Cit) pristine AgNPs, their effects on the cellular viability of the BeWo cells and their transport across the BeWo call layer were significantly lower. Upon placental exposure to Ag_2_S NPs, a relatively high concentration of total Ag in the cellular compartment was detected after 4 h exposure which remained unchanged upon longer exposure up to 24 h. This was in contrast with the time dependent behavior of the pristine AgNPs. Also, the lowest amount of total Ag, in comparison with the pristine AgNPs, was transported through the placental cell layer, even though the total Ag concentration in the cellular fraction was relatively high in comparison with the pristine AgNPs. Additionally, the aged particles did not induce any inhibition of the mESCs differentiation in the EST. The lower bioavailability of the Ag_2_S NPs could be explained by the sulfidation processes that these aged particles go through during their formation. The sulfidation of the AgNPs is likely to change the colloidal dynamics of these particles which increases the probability of these particles to aggregate and settle compared to the pristine NPs [10, 57, 58]. Possibly these aggregates bind strongly to the outer cell membrane, leading to high total Ag concentrations in the cellular fractions, but low transport through the cells. Taking these results into account, these aged AgNPs are imposing a very low risk for developmental toxicity. It is important to highlight that this study is one of the first studies that considered the potential hazards of aged AgNPs on the developing human fetus.

## Conclusion

To conclude, the AgNPs tested here were able to transport across BeWo b30 cell layer where the surface chemistry of these AgNPs influenced the amounts of AgNPs transported. The particles detected in the basolateral compartment, could result from transport of the original AgNPs or partly from de novo AgNPs formed from ionic silver that was transported. The observed inhibitory effects of the AgNPs on differentiation of mESCs were most likely the result of cytotoxicity rather than specific effects related to developmental toxicity. The aged AgNPs were significantly less cytotoxic and bioavailable and did also not induce in vitro inhibition of differentiation of mESCs. The combination of the BeWo placental transport model with the mESCs differentiation assay is considered a valuable alternative in vitro methodology for prenatal developmental toxicity screening and prioritization of silver nanoparticles.

## Methods

### Nanoparticles and chemicals

Negatively charged 20 nm ‘aged’ silver sulfide nanoparticles (Ag_2_S NPs) suspensions in milli-Q water were obtained from Applied Nanoparticles (Barcelona, Spain) with a mass concentration of 4.7 g/L. Three types of 50 nm silver nanoparticles with different surface modifications were purchased from Nanocomposix Inc. (San Diego, CA, USA); negatively charged lipoic acid BioPure (LA) AgNPs (pH = 6.1) suspended in milli-Q water, negatively charged citrate BioPure (Cit) AgNPs (pH = 7.4) suspended in 2 mM citrate buffer, and positively charged branched polyethylenimine (BPEI) BioPure AgNPs (pH = 7.0) suspended in milli-Q water. The silver mass concentration in the stock suspensions of the three AgNPs was 1 g/L. All AgNPs suspensions were stored at 4 °C in the dark. Silver nitrate (AgNO_3_) (Sigma Aldrich; St. Louis, MO, USA) was used as a control (source of Ag^+^) in all experiments. Dilutions of the AgNPs or AgNO_3_ were freshly prepared for every exposure experiment in complete cell culture media (depending on the cell line used in the experiment). 5-Fluorouracil (5-FU) was purchased from Sigma-Aldrich.

### Physicochemical characterization of nanoparticles

Hydrodynamic diameters of the AgNPs were determined using dynamic light scattering (DLS). Measurements were performed on samples containing 10 mg/L AgNPs suspended in nano-pure water using an ALV dynamic light scattering setup (ALV-Laser Vertriebsgesellschaft; Germany), consisting of a Thorn RFIB263KF photomultiplier detector, an ALV-SP/86 goniometer, an ALV 50/100/200/400/600 μm pinhole detection system, an ALV7002 external correlator, and a Cobolt Samba-300 DPSS laser. The measurements were performed immediately after preparation at room temperature. For each condition, samples were analyzed in triplicate; each measurement consisted of 10 technical replicate-measurements of 30 s each, at an angle of 90°. The results are expressed as the average hydrodynamic diameter (nm) ± standard deviation (SD) that was calculated using AfterALV software (AfterALV 1.0d, Dullware; USA).

The total silver content of the AgNPs suspensions and AgNO_3_ solution was analyzed using a NexION 350D inductively coupled plasma mass spectrometer (ICP-MS) (PerkinElmer, Waltham, MA, USA). Before analysis, samples were digested using an aqua-regia (1,3, 70% HNO_3_: 37% HCl) acid digestion for 30 min at 60 °C and diluted with nano-pure water. Silver was measured using the selected element-monitoring mode with *m/z* values of 107 and 109. A calibration curve of an ionic Ag standard (NIST-AgNO_3_) (Merck; Darmstadt, Germany) ranging from 0.1 to 50 μg/L was included. Rhodium (Merck) was used as an internal standard. The limit of detection (LOD_conc_) and limit of quantification (LOQ_conc_) were estimated to be 2 and 6 ng/L, respectively. The cell culture media (vehicle controls) did not contain detectable levels of Ag. All samples were analyzed in triplicate.

The particle sizes, size distributions, particle mass- and number-based concentrations of the AgNPs in the AgNPs suspensions and AgNO_3_ solution were quantified using single particle (sp) ICP-MS. The method for the spICP-MS measurements was described previously [59] . Briefly, the sample flow rate to the nebulizer was determined before the start of each series of measurements. The dwell time was set at 3 milliseconds and the total acquisition time was set at 60 s. A diluted suspension of 60 nm gold (Au) NPs (Nanocomposix) with a mass concentration of 50 ng/L was used before each analysis to verify the performance of the ICP-MS and to determine the transport efficiency. A calibration curve of ionic silver (NIST-AgNO_3_) with a concentration range of 0.1–20 μg/L was used for particle mass and size determination. The time scan data of the spICP-MS measurements were exported as .csv files, and the particle size, size distribution, and mass- and number-based concentrations were calculated from the spICP-MS data, using a dedicated spreadsheet. Details about the spreadsheet have been described previously [59]. The LOD_conc_ and LOQ_conc_ were estimated to be 20 and 67 ng/L, respectively. The NP size was calculated based on the particle mass, assuming spherical particles. The size detection limit (LOD_size_) was 20 nm and accordingly silver particles with sizes below this limit were included in the ionic silver fraction.

### Cell culture

ES-D3 adherent mouse embryonic multipotent stem cells (mESCs; ATCC; Wesel, Germany) were used at passage numbers between 4 and 12. The cells were cultured and maintained in 25 cm^2^ cell culture flasks (Corning; Oneonta, NY, USA) coated with 0.1% gelatin at 37 °C in a humidified 5% CO_2_ atmosphere in complete cell culture medium (DMEM^+^). DMEM^+^ was prepared by supplementing HyClone AdvanceSTEM Low Osmo Dulbecco’s modified Eagle’s medium (DMEM) culture medium (GE Healthcare Life Sciences; USA) with 20% (v/v) heat inactivated Fetal Bovine Serum (FBS) (ATCC; Manassas, VA, USA), 1% (v/v) Penicillin-Streptomycin-Glutamine 10,000 units penicillin, 10 mg streptomycin/mL, and 29.2 mg/mL L-glutamine (Gibco, Life Technologies; Paisley, UK). The cells were sub-cultured every 2–3 days using non-enzymatic cell dissociation solution (Sigma Aldrich) to detach the cells. The cells were maintained in an undifferentiated state by adding mouse leukemia inhibitory factor (mLIF; Sigma-Aldrich).

The adherent placental choriocarcinoma clone b30 (BeWo b30) was kindly provided by the Institute of Public Health of the Faculty of Health Sciences (University of Copenhagen, Denmark) with permission from Dr. Alan Schwartz (Washington University, St. Louis, MO) and confirmed to be mycoplasma free. The cells were used at passage numbers between 14 and 22. The cells were cultured and maintained in 75 cm^2^ cell culture flasks (Corning) at 37 °C in a humidified 5% CO_2_ atmosphere in complete cell culture medium (DMEM^+^). The DMEM^+^ was prepared by supplementing DMEM culture medium-GlutaMAX supplement-pyruvate (Gibco, Life Technologies) with 10% (v/v) heat inactivated FBS (Gibco, Life Technologies), 1% (v/v) Penicillin-Streptomycin 10,000 units penicillin, and 10 mg streptomycin/mL (Gibco, Life Technologies). The cells were sub-cultured every 3–4 days using trypsin-EDTA (Sigma Aldrich) to detach the cells.

### Cell viability

Cytotoxic effects of the AgNPs and AgNO_3_ were evaluated using the ATPlite luminescence assay system (PerkinElmer; Waltham MA, USA). In 96-well black flat bottom plates (Greiner bio-one; Frickenhausen Germany) each well was seeded with 100 μL of 1 × 10^5^ cells/mL BeWo b30 cell suspension in DMEM^+^. Plates were incubated at 37 °C and 5% CO_2_ for 24 h. The attached cells were then washed with 100 μL/well pre-warmed HBSS buffer w/o phenol red and exposed to 100 μL/well of freshly prepared dilutions (0.1–100 mg/L) of AgNPs or AgNO_3_. After 24 h exposure, the exposure medium was aspirated and 50 μL/well of mammalian cell lysis solution were added and the plates were shaken (700 rpm) for 5 min at room temperature in an orbital shaker (Heidolph-Trimax 1000; Schwabach, Germany). Next, 50 μL/well of substrate solution were added and the plates were shaken (700 rpm) for 5 min at room temperature and then incubated for 10 min in the dark at room temperature. The luminescence was then measured using a plate reader (BioTek Synergy™ HT Multi-Mode Microplate reader; Winooski VT, USA). The cell viability was expressed as percentage of the control. DMEM^+^ was used as a negative control and Triton-X100 (0.25%) (Sigma-Aldrich) was used as a positive control.

### BeWo b30 placental cell layer barrier integrity assessment

BeWo b30 cells were grown at a density of 1 × 10^4^ cells/cm^2^ on the upper side of transwell polycarbonate inserts (3 μm pore size, 1.12 cm^2^ surface area) (Corning) for 6 days (based on [16]. The integrity of the cell layer was assessed before exposure by measuring the transepithelial electrical resistance (TEER) using a Millicell ERS-2 Epithelial Volt- Ohm Meter (Millipore; Darmstadt, Germany). On day 6 post-seeding, only inserts with TEER values between 80 and 100 Ω.cm^2^ were used for further experiments. The TEER was also measured after exposure to confirm the barrier integrity and comparability of the TEER values before and after exposure.

Additionally, the cell layer integrity was evaluated before exposure to AgNPs suspensions and AgNO_3_ solution by measuring the transport efficacy of three different markers namely; lucifer yellow (LY) (Sigma-Aldrich) and low (4 kDa) and high (10 kDa) molecular weight fluorescein isothiocyanate dextrans (FITC-D) (Sigma-Aldrich). To the apical compartment, 500 μL/insert of 1 mg/mL of each of the integrity markers in DMEM^+^ were added separately. After 1 h incubation at 37 °C, the basolateral medium was collected, and the transport of the markers was determined by measuring the fluorescence at 485/530 nm using a fluorescence plate reader (BioTek Synergy™ HT Multi-Mode Microplate reader) and expressed as a percentage of the exposure concentration.

### BeWo b30 cellular uptake/association and transport of AgNPs and AgNO_3_

Six-day old BeWo b30 cell layer were exposed apically to 500 μL/insert of 1 mg/L of the AgNPs suspensions or the AgNO_3_ solution for 4, 6, 18, and 24 h at 37 °C and 5% CO_2_. Then, the media from the apical and basolateral compartments were collected. The cells were collected by trypsinzation (500 μL) and sonication (40 kHz for 15–20 min) to form cell lysate. The total silver content in all samples (apical, basolateral and cell lysate) was analyzed using ICP-MS. The particle size, size distribution, and mass- and number-based concentration in in all samples (apical, basolateral and cell lysate) after 24 h exposure using spICP-MS. The total Ag mass balance in the placental transport model upon exposure to AgNPs and AgNO_3_ was > 90%.

### In vitro developmental toxicity assessment of AgNPs and AgNO_3_

For the assessment of cell viability of mESCs used in the EST, the potential cytotoxicity of AgNPs and AgNO_3_ was evaluated after 24 and 120 h exposure, reflecting the shortest and longest exposure time during the EST. Each well was seeded with 100 μL of a 2 × 10^4^ (for the 24 h exposure) or 1 × 10^3^ cells/mL (for the 120 h exposure) cell suspension in DMEM^+^ without mLIF in 96-well black flat bottom plates (Greiner bio-one). The viability was assessed as mentioned above.

The potential of the AgNPs and AgNO_3_ to inhibit mESCs differentiation into beating cardiomyocytes was evaluated using the EST. The cells were exposed from day 3 to 10 of the 10-day mESCs differentiation. The wells of the 96-well plate (Corning) were filled with 200 μL/well PBS. To start the assay hanging droplets (20 μL) of a 3.75 × 10^4^ cells/mL mESCs suspension were placed on the inner side of a lid of a 96-well plate. Sterile lids of Eppendorf tubes were placed on each corner of the 96-well plate lid to avoid contact of the droplets with the plate. Then the plate was sealed with Micropore tape (3 M; Germany) to prevent evaporation of the hanging drops. The plates were incubated for three days at 37 °C and 5% CO_2_ in a humidified atmosphere to allow the formation of embryoid bodies (EBs). On day 3, the formed EBs were transferred to 6 cm non-treated tissue culture petri dishes (Greiner bio-one) with 5 mL of DMEM^+^ containing AgNPs or AgNO_3_ and incubated at 37 °C and 5% CO_2_ in a humidified atmosphere for 2 days to allow growth of the EBs. A concentration range between 0.1–25 mg/L was used for each type of AgNPs, while for AgNO_3_ a concentration range between 0.05–5 mg/L was used. DMEM^+^ was used as a negative control and 1 μM 5-FU was used as a positive control. On day 5, the EBs were transferred to a 24-well plate (Corning) where each well contained one EB in 1 mL of the same concentration of AgNPs or AgNO_3_. On day 10, using a light microscope, the wells were visually inspected for contracting cardiomyocytes. The number of wells/plate containing contracting cardiomyocytes were recorded where the experiment was considered valid if at least 21 of the 24 wells of the negative control sample contained contracting cardiomyocytes. For each concentration of each treatment, the fraction of successfully differentiated EBs into contracting cardiomyocytes was calculated and expressed as percentages of the number of wells with beating cardiomyocytes from the number of wells initially seeded with EBs for each concentration.

### Characterization of AgNPs dissolution in DMEM^+^

The stability and dissolution properties of the four AgNPs were evaluated in 1 mg/L AgNPs suspensions in DMEM^+^ upon incubation in the dark at 37 °C. At 0 and 120 h, aliquots from each AgNPs suspension were extracted for analysis. spICP-MS was used to quantify the total Ag content, particle size, size distribution, and mass- and number-based concentration in all samples as described earlier.

### Confocal microscopy of differentiated cardiomyocytes

For confocal imaging, the EBs formed in the EST were transferred into 8 - well μ-Slides (Ibidi; Gräfelfing, Germany) for exposure and differentiation into cardiomyocytes. The EBs were exposed to the AgNPs suspensions or AgNO_3_ solution using similar conditions as used in the EST. After 5 days exposure, the exposure medium was discarded, and the cells were fixed with 4% paraformaldehyde (Sigma-Aldrich) for 15 min at room temperature. The cells were washed 3 times with PBS for 5 min after discarding the fixation solution. The cells were permeabilized with 0.25% Triton X-100/PBS for 15 min at room temperature. The cells were then washed again 3 times with PBS for 5 min before incubating them with the blocking buffer (1%BSA in PBS) for 30 min at room temperature. After discarding the blocking buffer, Phalloidin - Alexa 488 (6 units) (Dyomics; Jena, Germany) was added to stain cellular actin and the cells were incubated for 30 min at room temperature. Cells were washed three times with PBS before incubating the cells for 10 min at room temperature with RedDot-2 (1: 200) (Biotium; Fremont, CA, USA) to stain the nuclei. Finally, the cells were washed with PBS and stored in the dark until analysis. The cells were analyzed using a confocal laser scanning microscope (SP5X-SMD; Leica Microsystems, Wetzlar, Germany). Samples were excited with 665 and 495 nm lasers and backscattered light was used to detect AgNPs using a 543 nm laser.

### Statistical analysis

Each data point represents the average of three replicates (*n* = 3) and the results are shown as mean ± standard deviation. Prism (v.8.0.1; GraphPad, USA) software was used for statistical analysis using a one-way analysis of variance (ANOVA) with a Bonferroni’s post-test. A *p-value* < 0.05 was considered significant.

## Supplementary information

**Additional file 1.** Supplementary information accompanies this paper

## Data Availability

The datasets used and/or analyzed during the current study are available from the corresponding author on reasonable request.
