# Supplementary material for: Combination of the BeWo b30 placental transport model and the embryonic stem cell test to assess the potential developmental toxicity of silver nanoparticles
Source: Part Fibre Toxicol. 2020 Mar 10;17:11. doi: 10.1186/s12989-020-00342-6 (PMC7063716; doi:10.1186/s12989-020-00342-6)
Supplement: Supplementary file 1 — Additional file 1. Supplementary information accompanies this paper [file 12989_2020_342_MOESM1_ESM.docx]

# Supplemental online material

**Combination of the BeWo b30 placental transport model and the embryonic stem cell test to assess the potential developmental toxicity of silver nanoparticles**

*Ashraf Abdelkhaliq^1,2,3^, Meike van der Zande^2^, Ruud J.B. Peters^2^, and Hans Bouwmeester^1^*

^1^ Division of Toxicology, Wageningen University, P.O. box 8000, 6700 EA, Wageningen, the Netherlands

^2^ Wageningen Food Safety Research (WFSR) (Previously: RIKILT - Wageningen Research) – part of Wageningen University and Research, P.O. Box 230, 6700 AE, Wageningen, the Netherlands

^3^ Food Science and Technology Department, Faculty of Agriculture – Alexandria University, Alexandria, Egypt

**Keywords:** silver nanoparticles, surface chemistry, placental transport, embryotoxicity, single particle-ICP-MS


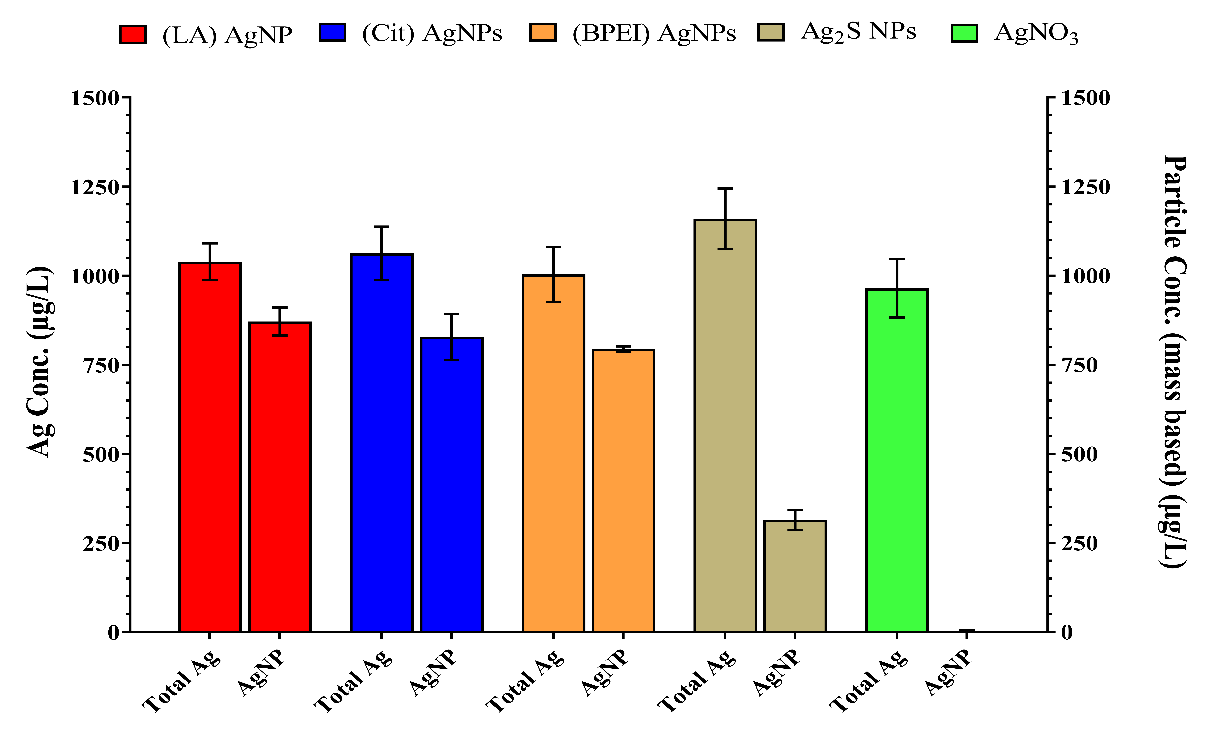


***Figure S 1:*** *Silver content in 1 mg/L AgNPs suspension and AgNO_3_ solution expressed as* *total Ag and as AgNPs measured using ICPMS sp-ICPMS, respectively. Concentrations are given as the mean ± SD (n=3). No AgNPs were detected in the AgNO3 solution (< LOD).*

**C)**

**A)**

**B)**











**D)**

***Figure*** ***S2:*** *Number-weighted size distributions of AgNPs generated by spICP-MS measurements of DMEM suspensions of* ***A)*** *(LA) AgNPs,* ***B)*** *(Cit) AgNPs,* ***C)*** *(BPEI) AgNPs and* ***D)*** *Ag_2_S NPs.*
